# Allergens from Edible Insects: Cross-reactivity and Effects of Processing

**DOI:** 10.1007/s11882-021-01012-z

**Published:** 2021-05-30

**Authors:** Laura De Marchi, Andrea Wangorsch, Gianni Zoccatelli

**Affiliations:** 1 Department of Biotechnology, University of Verona, Verona, Italy; 2 Molecular Allergology, Paul-Ehrlich-Institut, Langen, Germany

**Keywords:** Edible insects, Food allergy, Tropomyosin, Arginine kinase, Cross-reactivity, Food processing

## Abstract

**Purpose of Review:**

The recent introduction of edible insects in Western countries has raised concerns about their safety in terms of allergenic reactions. The characterization of insect allergens, the sensitization and cross-reactivity mechanisms, and the effects of food processing represent crucial information for risk assessment.

**Recent Findings:**

Allergic reactions to different insects and cross-reactivity with crustacean and inhalant allergens have been described, with the identification of new IgE-binding proteins besides well-known pan-allergens. Depending on the route of sensitization, different potential allergens seem to be involved. Food processing may affect the solubility and the immunoreactivity of insect allergens, with results depending on species and type of proteins. Chemical/enzymatic hydrolysis, in some cases, abolishes immunoreactivity.

**Summary:**

More studies based on subjects with a confirmed insect allergy are necessary to identify major and minor allergens and the role of the route of sensitization. The effects of processing need to be further investigated to assess the risk associated with the ingestion of insect-containing food products**.**

## Introduction

The Food and Agriculture Organization of the United Nations estimated that by 2050, the world’s population would reach about 9 billion people [1, 2], raising concerns about the capacity to feed such a large population. To solve this problem, alternative and more sustainable food sources from the economic and environmental points of view are necessary.

In this scenario, insects represent one of the most promising solutions. Insects are considered a source of nutrients like polyunsaturated fatty acids, essential amino acids, micronutrients, and protein [3]. In addition, entomophagy might have various positive implications in terms of sustainability. Indeed, in comparison to livestock, breeding insect produces lower greenhouse gas emissions and water pollution and is characterized by higher feed conversion efficiency and lower land dependency [1, 2]. Furthermore, it is possible to utilize several plants and organic wastes as feed [4–6].

However, some risks may stem from the consumption of insects, essentially due to possible chemical (e.g., heavy metals accumulation) and microbiological contaminations. Some insects have also been reported to cause allergic reactions through inhalation, direct contact, sting/bite, and also by ingestion [2, 4, 7–9]. The adverse reactions described after the ingestion of insects can be caused by cross-reactivity with other taxonomically related food allergens like crustaceans [10–12, 13••], but also with inhalant allergens such as house dust mites (HDM) [14]. The rationale is based on the presence of common allergens among invertebrates, like tropomyosin (TM), arginine kinase (AK), and glyceraldehyde 3-phosphate dehydrogenase (GAPDH) [14, 15].

Allergic reactions to edible insects have been mainly described in Asian [16–19] and in African [20–22] countries, where entomophagy is a habitual practice. In some cases, the reactions occurred in non-atopic subjects, suggesting that the mechanism was based on primary sensitization to insect allergens [23, 24].

To hide the unappealing nature of eating whole insects, in Europe and in general in Western countries, these are mainly employed as ingredients to enrich fortified products. This poses interesting questions about the possible effects that different technological processes and food matrices (starch, proteins, etc.) may have on IgE-binding epitopes and how this could influence their susceptibility to gastrointestinal digestion.

Basing on research articles, clinical, and case reports, this review aims to (1) analyze the cross-reactivity of edible insects with other food and inhalant/indoor allergens, (2) evaluate the role of primary sensitization, and (3) characterize the effects that food processing might have on main cross-reactive allergens.

## Novel Foods and EU Legislation

**Fig. 1:**
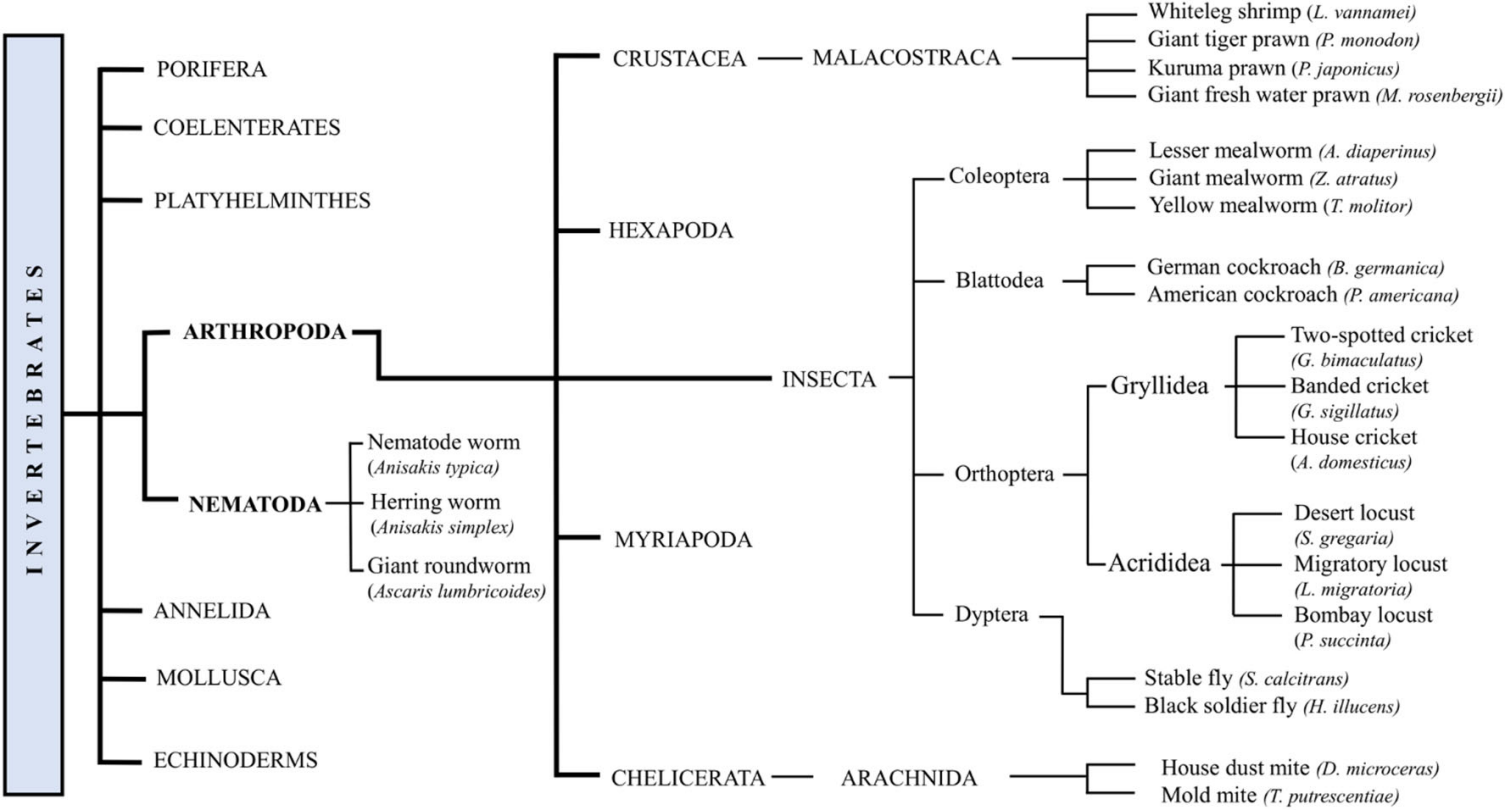
Simplified classification of the insect species discussed in the present review

Edible insects are considered novel foods, i.e., foods that had not been consumed to a significant degree by humans in the EU before 15 May 1997. In 2018, the latest EU regulation on novel foods came into force, with the result that all the novel foods need to follow a centralized approval system, which comprises a complete risk assessment, performed by the European Food Safety Agency (EFSA), including the allergenic risks. Due to different interpretations of previous EU legislation dealing with foods and novel foods (EU REG 258/97 and 1169/2011) by European countries [25, 26], products containing edible insects were already present on the markets of some member states (Belgium, UK, the Netherland, and Denmark) before 2018. With the latest regulation, it was clarified that all the products containing edible insects which were already on the market might continue to be sold until they are approved through the new centralized procedure, but not later than January 2020. This authorization has been recently extended [27•]. Until now, the following insect species are present in the summary of applications to the European Commission: house cricket (*Acheta domesticus*) as powder or ground form, lesser mealworm (*Alphitobius diaperinus*) as whole and ground larvae products, banded crickets (*Gryllodes sigillatus*) in dried form, migratory locust (*Locusta migratoria*) as whole and ground insect, and dried yellow mealworm (*Tenebrio molitor*) in larval or adult stage. It can be observed that the families of *Gryllidae* (cricket) and *Tenebrionidae* (mealworm) seem to attract the major interest of the food industries. Figure 1 shows a summarized classification of the species discussed in the present review.

## Cross-reactivity and Primary Sensitization

Many proteins have been discussed to play a role in the elicitation of allergic reactions after the ingestion of edible insects [7], and, according to Barre and collaborators [28], there are pan-allergens widespread in invertebrate groups which belong to a limited number of protein families. The reviewed literature relates to two main topics: the characterization of potential insect allergens compared to the already well-known allergens using allergic patients’ sera and the route of sensitization to verify whether insect allergens may act as primary sensitizers.

### Cross-reactivity Between Insects and Other Arthropods: General Overview

As presented in the database of allergen families (AllFam database, http://www.meduniwien.ac.at/allfam), the most important and widely described insect allergen is tropomyosin [29], a protein-based on an alpha-helical structure involved in muscular contraction. The most relevant source of TM, as an officially accepted allergen, is represented by shellfish, i.e., crustaceans, mollusks, and cephalopods (see Pedrosa et al. [30] as a representative review), but also parasites/nematodes, like the herring worm *Anisakis simplex* or common roundworms *Ascaris lumbricoides*, contain TM described and accepted as food allergens, e.g., Ani s 3 [31] and Asc l 3 [32]. Up to now, 16 arthropod TM have been registered as food allergens, according to WHO/IUIS allergen nomenclature subcommittee. Tropomyosin is also a very common and widespread inhalant allergen, with cockroaches and mites (e.g., house dust mites and storage mites) as major sources [33, 34, 35•, 36]. All known TM share a common three-dimensional structure and are characterized by high amino acid (AA)-sequence identity. The other crucial invertebrate-pan allergen is arginine kinase, a protein with enzymatic function and a highly conserved amino acid sequence among various invertebrate species [28] characterized by a β-sheet domain surrounded by α-helices [37]. Up to now, according to the WHO-IUIS, AK from 13 species have been accepted as allergens (6 as airborne allergens and 7 as food ones), ranging from mites (Der f 20, Der p 20, Tyr p 20) to cockroaches (Bla g 9, Per a 9) up to shellfish (Lit v 2 and Pen m 2) and moths (Plo i 1). Besides these two major allergens, several potentially clinically relevant allergenic proteins from edible insects are described in the literature. In many studies, in vitro assays (e.g., immunoblotting, BAT) have been performed to analyze the cross-reactivity between insect proteins (major or minor allergens) and already well-known allergens, in most of the cases using sera of patients allergic to shrimp, mites, or other invertebrates. The co-sensitization to yellow mealworm (YMW) in crustacean allergic patients has been widely demonstrated [14, 38]. An extensive study on the potential allergenic YMW proteins by Barre et al. [39•], including a shrimp allergic population and a second group of HDM allergic subjects with confirmed Der p 10 sensitization, revealed a high number of potentially allergenic proteins in *Tenebrio molitor*. In addition to the already discussed α-amylase [14], AK [14], TM [14, 40], and heat shock protein (HSP) 70 [14], apolipophorin-III (apoLp-III), larval cuticle protein (LCP), and a 12-kDa hemolymph protein were detected by the IgE of shrimp allergic patients [39•]. Apolipophorins until now have only been described as inhalant allergens from mites. It is still a matter of debate whether the primary inhalant allergy to invertebrates could lead to a so-called secondary food allergy, as already described for the pollen-food syndrome [35•].

Besides the pan-allergen AK, other allergens were identified in silkworm (*Bombix mori*): Jeong and collaborators [41] described a 27-kDa glycoprotein as a possible allergenic protein, though IgE reactivity using silkworm allergic patients sera was low. Zhao et al. [19] indicated chitinase and paramyosin as two other potential allergens. Chitinase has already been discussed as an allergen between different species, especially in plant kingdom, like fruits [42]. AK from silkworm (SW) is of particular relevance because it is the only allergen (Bomb m 1) from edible insects officially accepted as food allergen (www.allergen.org) and included in the IUIS allergen database. Bomb m 1 was introduced into the database in 2010 by Liu and colleagues [43], who showed that 25% of patients claiming allergic symptoms to SW or silk products were positive by skin prick test (SPT) to crude extracts from SW. All ten patients were sensitized to recombinant Bomb m 1 [43]. Although Liu et al. did not clearly define the route of exposure leading to SW allergy in this patient cohort, Bomb m 1 was accepted as a food allergen, as SW is commonly used in traditional Asian cuisine and can lead to anaphylactic reactions [16, 44].

Very few studies were carried out on cricket proteins’ cross-reactivity in patients allergic to shellfish or HDM. AK and hexamerin 1B (HEX1B) were identified in *Gryllus bimaculatus* by Srinroch and collaborators [15] as major and minor allergens, respectively, using prawn allergic patient’ sera. However, Kamemura et al. [13••] indicated TM as a cross-reactive allergen between shrimp and crickets. In their study, they performed immunoblotting and a competitive inhibition ELISA using sera from 9 subjects allergic to shrimp, showing that a cricket protein of 40 kDa (identified as a high molecular weight tropomyosin isoform) reacts with shrimp-allergic patients sera [13••]. However, due to the limited sensitivity of the method, the authors did not exclude the presence of other cross-reactive allergens. The study of Pali-Scholl et al. [45••] not only confirmed the cross-reactivity between crustaceans and cricket (*Acheta domesticus*) proteins but showed that crustacean-, HDM-, and stable flies-allergic patients cross-recognize cricket proteins. Other possible insects that could lead to allergic cross-reaction in crustacean-, HDM-, and flies-allergic patients are locusts (*Locusta migratoria* and *Schistocerca gregaria*), as suggested by Pali-Scholl in the same work [45••].

### Sequence Analysis

Besides using immunoenzymatic methods, the analysis of sequence identity or homology between insect proteins and the already well-characterized food allergens (e.g., from crustaceans) gives the possibility to infer common properties shared by allergenic proteins and maybe predict the allergenic potential [46]. Although at the moment there is no clue about the structural characteristics underlying allergenicity [42], this approach is part of the risk assessment procedure indicated by EFSA for novel food proteins. Indeed, no validated or predictive method for the assessment of allergenicity of a novel protein or protein-containing product is globally recognized. For this reason, for the latest novel foods submissions and the preliminary safety evaluation of novel proteins, parts of the GMO allergenicity risk assessment guidelines drafted by EFSA have been used [47]. This procedure, the so-called weight-of-evidence approach, is based on an integrated case-by-case approach. The WHO guidelines for predicting allergenic cross-reactivity suggest a threshold of 35% of sequence identity to a known allergen using a sliding window of 80 amino acids [48•], or a complete identity within an 8 amino acid peptide [49]. An example of allergenicity assumption through sequence alignment analysis is the study of Liu and collaborators [43] on *Bombix mori* AK. Due to the significant similarity (ranging from 81 to 92%) shared with the other AK associated with allergenic reactions and the extremely low e-values resulting from the comparison, particularly with Plo I 1 and Pen m 2, it can be concluded that AK of *Bombix mori* possesses allergenic potency.

The high sequence identity and the already extensively investigated IgE-cross reactivity between TM, AK, and other allergens from different species, like shellfish, cockroach, mites, and parasites, lead to the assumption that the same group of proteins share cross-reactive IgE-epitopes, capable of inducing allergic reactions upon consumption of insects. However, cross-reactivity seems not to be systematic. Francis et al. [50••] investigated the cross-reactivity of AK from *A. domesticus* and *T. molitor* in subjects exposed to edible insects, and no cross-reaction phenomena were observed, although AK from different insects are acknowledged to share high sequence identity (70% on average) and homology (90% on average) [37]. Small differences in the sequences could likely cause a differential IgE binding, as Palmer and collaborators also stated [51••]. This could be the reason for the absence of cross-reactivity observed by Francis and co-workers [50••].

It has to be taken into consideration that, although the amino acid identity between two compared structures correlates with the probability of cross-reactivity, the mere amino acid identity and the structural homology are weak predictors and should be used prudently [48•, 51••]. Thus, EFSA guidelines suggest additional clinical tests, like SPT or basophils activation test (BAT), and the golden standard to determine the absence/presence of allergenicity, i.e., the double-blind placebo-controlled food challenge (DBPCFC) [47, 52]. In general, data on allergenic risk due to insect consumption are very limited, as most of the trials have been conducted with a scarce number of participants (*n*<20) [53]. To our knowledge, the only complete allergenicity study on edible insects is the one conducted by Broekman and collaborators [52]. The study was performed on a group of 15 shrimp allergic patients characterized by SPT, BAT, and immunoblotting and subsequently included in a DBCPFC trial with blanched YMW (*T. molitor*). None of the patients knowingly consumed YMW proteins. The results achieved showed that 14 out of 15 patients were sensitized to YMW TM and/or AK, and 13 out of 15 reacted positively to the oral test. Eleven patients out of 12 were sensitized to TM of other species, like Anisakis, HDM, and cockroach, and to Bomb m 1, probably due to the proteins’ high sequence identity. This result contributed to the complete risk evaluation of dried YMW as a novel food, published on 12 January 2021 by EFSA scientific network [54]. The expert panel concluded that the insect is safe under the proposed uses and use levels; however, in the proposed conditions, the ingestion may induce primary sensitization and allergic reactions or cause cross-reactivity phenomena in subjects allergic to crustaceans and HDM. This publication is the first complete evaluation at the European level on an edible insect as a novel food. Thus, the dried YMW will be likely be included in the European Novel food catalog.

### Need of Standardization

An important aspect that has to be considered is the fact that most of the studies based on immunoblotting, BAT, and/or ELISA techniques described the sensitization of patients allergic to shellfish/inhalant allergens (e.g., cockroach or mites) to protein extracts derived from edible insects [11, 13••, 15, 45••, 51••], and in some cases also to purified proteins [50••]. As claimed by Broekman et al. [55], particular attention should be paid to the extract preparation because this could modify the representative set of proteins. Indeed, Verhoeckx et al. [14] demonstrated that using Tris buffer or urea solutions, different sets of proteins could be extracted from YMW. Due to this variability, there is a great need for studies that include SPT or DBPCFC to confirm insect allergic patients and to prove that proteins expressed in edible insects, independently if already described as allergens or belonging to a completely new protein family, can elicit allergic reactions and not only showing IgE-cross-reactivity (sensitization) without causing symptoms.

## Primary Sensitization

A question still open is whether the type of primary sensitization route is important for the IgE-cross reactivity of pan-allergens. Allergens from the same protein groups are accepted as allergens, for example, from shellfish and HDM or cockroaches. However, shellfish and crustacean allergens are real food allergens, as they lead to sensitization via the ingestion route, while mite and cockroach allergies are caused by the sensitization through inhalation of allergenic proteins. Although it is well known that pollen allergens, acting as a primary sensitizer, could lead to a secondary food allergy (e.g., pollen-fruit syndrome based on pathogenesis-related proteins 10 (PR-10), profilins, and lipid transfer proteins (PR-14) [56]), it is not necessarily true for other allergen families, like tropomyosins or arginine kinases. Using patient groups without clear allergic reactions to edible insects does not help to understand whether the latter can act as primary sensitizers. To our knowledge, the only study showing the sensitizing capacity of insects was carried out employing mice and administering by gavage a YMW extract in combination with cholera toxin as adjuvant [57]. In this study, Broekman and collaborators found IgE against several proteins in 2 out of 6 mice tested, such as larval cuticle protein (A1A, A2B, A3A), TM, actin, and AK. Unfortunately, other sensitization studies performed in rats and guinea pigs did not return statistically relevant results [58–60]. Barre et al. [39•] could show that 95% (20/21) of patients with anaphylaxis to shrimp are sensitized to YMW extract, whereas only 15% (2/13) of Der p 10 positive HDM-allergic patients showed sensitization to YMW. Even if the mite allergic patients were preselected to Der p 10, and tropomyosin could clearly be detected in the YMW extract via mass spectrometry (MS) analysis, the sensitization to tropomyosin was very low. Similar findings were published by van Broekhoven et al. [40], where a pool of sera of patients allergic to HDM did not recognize TM of three different MW species extracts. In contrast, crustacean allergic patients clearly showed sensitization to tropomyosin in the same extracts. The identity degree between Der p 10 and YMW TM and between Lit v 1 and YMW TM are very similar, i.e., 66 and 70% respectively (https://blast.ncbi.nlm.nih.gov), but differences in crucial sequences of epitopes regions might explain the differences observed in terms of cross-reactivity, as suggested Palmer and co-workers [51••].

Occupational allergies should also be considered in this scenario. It is known that SW and mealworm breeders, as well as people exposed to locusts, could raise inhalant allergy against other insects [61, 62••, 63–65]. Would they also react after eating edible insects? Broekman et al. [57] conducted an interesting study on subjects with a history of clinical symptoms after domestic or professional exposure to YMW and that as well consumed some insects. The results showed that a longer exposure period or the ingestion of higher doses is required to develop food allergy to mealworms. Moreover, since 3 out of 4 studied subjects showed higher levels of IgE to mealworms than to any other food or inhalant allergens they are allergic to, the authors considered that mealworms could act as the primary sensitizer, even if larval cuticular proteins, instead of TM and AK, seems to play the main role in primary mealworms allergy. In a further study, the same authors suggested the possibility that the sensitization to insects might be species-specific, meaning that allergies to different insects could be caused by specific proteins and thus mealworms-sensitized subjects are not supposed to react to all insects [11].

## Effect of Processing on Edible Insects’ Potential Allergens

As already said above, part of the risk assessment for novel foods includes the weight-of-evidence approach to prevent the introduction of an allergenic protein into a food source. However, this strategy is not applicable to predict the primary sensitization potency of a protein when there are no subjects with a history of sensitization to the target protein [47]. Another important consideration is that the allergenic potency of a food protein is probably influenced by factors such as food processing and the interaction with the matrix [66]. These factors are central in the case of edible insects since the most likely way of consumption in Western countries is in the form of ingredients to enrich processed foods.

Insects intended for food formulations are necessarily subjected to post-harvest processing, e.g., blanching, pasteurization, and sterilization [45••] to ensure their microbiological safety. It is well known that heat processing could affect the allergenic potency of proteins [67]. However, the effect is not predictable and could result in an enhancement of the potency, like in the case of peanut proteins, or in a reduction/elimination of the IgE-binding capacity, e.g., tree nut allergens [55, 66, 68]. Limited information concerning the effects of processing on the allergenicity of insect proteins is available, but due to the close taxonomic relationship of insects to shellfish, it can be assumed that the alterations should be similar to those observable on shellfish [10, 55]. Unfortunately, also studies on the effects of processing on shellfish (and in particular crustaceans) proteins are contradictory: some authors reported no significant differences in shrimp allergenicity after boiling [69, 70], while others observed an increased IgE-binding capacity [71–73]. However, the impact of treatment on the IgE-binding capacity does not necessarily correlate with clinical symptoms.

A few studies on the effect of different thermal treatments on edible insects (mealworms, SW, and locusts) have been conducted (see Table 1), and recently, also the impact of technological processes such as enzymatic hydrolysis has been investigated as a tool to reduce the allergenicity of different food matrices [45••, 74••, 76, 78••]. The processing methods applied in the case of mealworms affected the solubility of IgE-binding proteins without, in general, decreasing their immunoreactivity. This could be due to structural modifications and aggregation phenomena, as in the case of TM that is supposed to interact with the muscle protein matrix [55]. The heat treatment of SW reduced the immunoreactivity of IgE-binding proteins. These showed different digestion profiles depending on the enzymes employed. Proteins in the 25–33 kDa range displayed greater stability to heat treatments and digestion [74••].

**Table 1.** List of articles studying the effect of processing on edible insects

Insect species	Identified allergens	Patient group	Applied process	Outcome	Reference
Mealworm					
*T. molitor*	•Putative allergens soluble in water: cationic trypsin, TM, AK, actin, α-tubulin, β-tubulin, α-amylase, fructose-bisphosphate aldolase, ovalbumin-like protein, myosin light chain. •Urea fraction: cationic trypsin, TM, ovalbumin-like protein	No. 7 patients allergic to crustacean and HDM (sensitized to Der p 10). No. 15 negative controls	*Sample:* Mealworm protein extract *Process:* Static pepsin digestion (60 min)	•Water soluble proteins partially digested after 15 s, immunoreactivity decrease but not completely lost even at the end of digestion process. •Urea soluble proteins: 32-kDa band completely degraded after 10 min; 40 kDa band as water soluble proteins.	Verhoeckx et al. 2014 [14]
	Same as in Verhoeckx et al. 2014	No. 3 shrimp-allergic patients	Sample: Raw mealworms Processes: •blanching for 1 min at 100°C; •boiling in water for 10 min at 100°C; •baking for 3 to 5 min at 1000W •frying for 30 s at 180°C	•processing affected protein solubility (AK became less soluble in tris-buffer, TM more soluble in tris-buffer. •TM partially degraded after processing, but still immunoreactive •Modification of solubility: TM and MLC were more soluble after heat processing, while solubility of AK decreased. •the processing did not reduce IgE-binding capacity and IgE cross-linking functionality of mealworm allergens	Broekman et al. 2015 [55]
*Tenebrio molitor*, *Zophobas atratus*, *Alphitobius diapernus*	•Crustacean allergic patients: actin, TM •HDM allergic patients: paramyosin, α-amylase, actin, larval cuticular protein, HEX1B precursor, myosin	No. 6 crustacean-allergic patients; No. 7 HDM-allergic patients (not to crustaceans). No. 6 non-atopic subjects	Sample: Frozen larvae Processes: •Lyophilization (−50 °C, 150 Pa) •Boiling (5 min) •Frying (5 min, 180 °C) •Gastrointestinal simulated digestion	•Crustacean allergic patients’ IgE recognized mealworm proteins in all processed samples but not in fried sample. After digestion, proteins were still detectable in lyophilized and boiled, but not in fried sample. •Most proteins were immunoreactive to HDM allergic patients IgE after processing; after digestion, only 25 kDa protein from *Z. atratus* lyophilized sample was immunoreactive.	van Broekhoven et al. 2016 [40]
Silkworm					
*B. mori*	Proteins with molecular weight between 25 and 33 kDa (putative 27–kDa glycoprotein	no.15 patients allergic to silkworm pupae	*Sample:* Silkworm pupa protein extract		He et al., 2020 [74••]
			*Processes:*		He et al. 2020 [74••]
			•heat treatment at 20, 40, 60, 80, 100, and 120°C for 20 min each	Heating above 60°C (especially at 120°C), significantly decreased the allergenicity; putative glycoprotein showed heat resistance below 100°C.	
			•Simulated digestion: Pepsin digestion (150 min), trypsin digestion (150 min), pepsin-trypsin digestion	•> 33-kDa protein gradually degraded and then vanished after 120 min of pepsin treatment; stable to trypsin digestion • 25–33 kDa proteins stable to pepsin digestion but degraded after tryptic digestion	
			Acid-alkali treatment	•protein stable at neutral pH, degradation at low pH	
Locust					
*Patanga succincta*	HEX, enolase, AK, pyruvate kinase^a^, GAPDH^a^	No. 16 prawn-allergic patients	*Sample:* Frozen whole insect. *Process:* Frying at 108.80±5.78°C for 3min	•Enolase and HEX showed reduced immunoreactivity, •AK showed significant allergenicity decrease •Immunoreactivity of GAPDH and pyruvate kinase increased	Phiriyangkul et al. 2015 [75]
*Locusta migratoria*	Putative α-amylase	No. 3 crustacean-allergic patients No. 8 HDM-allergic patients	*Sample:* Basic protein extraction from freeze-dried and blended locust without wings and legs *Processes:* •enzymatic hydrolysis (alkalase, neutrase, flavourzyme, papain; 50°C, pH 7, for 2 h) •heat treatment (80–100° for 10 min; 121–138°C for 20 min)	IgE-binding capacity lost after enzymatic hydrolysis or heat treatment of the sample.	Pali-Schöll et al. 2019 [45••]
Cricket					
*Gryllodes sigillatus*	TM	No. 10 shrimp-allergic sera	*Sample:* Whole crickets *Process:* Alcalase hydrolysis	Degree of hydrolysis (DH) influences the IgE binding capacity: -decreased (DH 15–40%), -unchanged (DH 52%) -eliminated (DH 50–85%)	Hall et al. 2018 [76]
*Acheta domesticus*	TM	No. 20 shrimp-allergic sera	*Sample:* grinded crickets *Processes:* •Baking for 10 min at 180 °C in a model bakery product (biscuit containing 10% *A. domesticus*) •Static simulated digestion: pepsin digestion (60 min) + pancreatin digestion (120 min)	•TM stable to baking process •TM of grinded crickets immunoreactive after simulated digestion, despite a partial degradation. TM from enriched biscuits was more stable to pepsin digestion but almost lost its immunoreactivity during pancreatin digestion.	De Marchi et al., in press [77••]
Black soldier fly					
*Hermetia Illucens*	TM	Crustacean-allergic patients (sensitized to Pen a 1)	*Sample:* Grinded flies after freezing *Process:* Enzymatic hydrolysis with protease from *Bacillus licheniformis*	Protein hydrolysate is still reactive toward IgE	Leni et al. 2020 [78••]

^a^Protein identified only in fried sample

The immunoreactivity of migratory locust was lost after severe heat treatments or by enzymatic hydrolysis [45••]. The frying treatment of *Patanga succincta*, another kind of locust, reduced the immunoreactivity of AK, enolase, and HEX, while GAPDH and pyruvate kinase showed higher reactivity [75]. With respect to crickets, an enzymatic treatment with alcalase abolished the relativity of TM in homogenized *Gryllodes sigillatus* [76]*.* The IgE-binding capacity of *Acheta domesticus* TM was still immunoreactive after simulated digestion. The baking process of a model enriched food improved the stability of TM in the gastric phase [77••].

The treatment of black soldier fly by a protease from *Bacillus licheniformis* to reduce the allergenicity did not abolish the immunoreactivity of the proteins [78••].

Studies that use protein extracts cannot be considered exhaustive since heating a food matrix (e.g., the whole shrimp muscle or a complex food matrix) has reasonably different effects compared to the treatment of water extracts or purified proteins. Indeed, other food components may interact with the allergens changing their solubility and structure and thus affecting IgE epitopes. The consumption of insects mainly occurs in eastern countries, where they are usually ingested as raw or after a simple food processing, such as frying and boiling. Therefore, the majority of the allergenicity studies focused on these processed forms. Differently, insects have been introduced in Western countries as ingredients of enriched foods, such as snacks [79, 80], pasta [81], and meat preparations like sausages [82] in order to be more acceptable by the consumer. For the preparation of many industrial products several food processing methods are used, e.g., different drying methods, ultra-high temperature (UHT), and short-time pasteurization [66]. One of the most convenient, productive, and cost-effective ways to produce snacks is by extrusion. This technology utilizes a single screw or a set of screws to force mixed food ingredients through a small opening. During the process, foods are cooked, and the setting of the conditions allows to obtain a final product with a precise shape and to increase characteristics like solubility, viscosity, or swelling power [83]. Evidence that this process can decrease the allergenic potency of legumes, for instance, is available. Indeed, Franck et al. [84] indicated that specific soybean allergens lost their reactivity after the texturization of soybean by extrusion. Recently, Zheng and collaborators [85•] studied the effect of extrusion on soybean and corn meal proteins concluding that all the extruded proteins seem to show a lower immunoreactivity compared to the raw materials. Moreover, the circular dichroism analysis of the proteins showed that the processing leads to a structural conformation shift to a more β-strand-based structure [85•]. This evidence may have important implications in the reduction of the allergenic potency of insect proteins incorporated into extruded formulations. TM possesses a characteristic alfa-helical structure that may be affected by the extrusion process. Furthermore, the addition of other starchy ingredients like cereal or legume flours, commonly employed in this process, may lead to the establishment of interactions between the insect proteins and the matrix, differently impacting on the epitopes. We think that this hypothesis should be further investigated.

Food matrix may affect not only the allergenic potency of a protein but also its susceptibility to digestion. In fact, Schulten and collaborators [86] investigated whether the presence of a food matrix could influence the absorption of model allergens (from cow milk and hazelnut) after gastrointestinal digestion. The group concluded that a food matrix rich in protein and carbohydrates could hamper the degradation of allergens during the digestion process. Van Broekhoven et al. [40] studied the effect of processing and in vitro digestion on allergic cross-reactivity of MW extract in HDM allergic patients. Proteins underwent boiling, frying, and lyophilization. The major allergen (TM) seems to be stable to boiling and to in vitro digestion, while the allergenicity seems to be decreased after frying, which would confirm that the type of processing method influences the allergenic potency of an allergen [40].

Another important consideration about the incorporation of insect derivates in food preparations concerns the effective amount of insect protein resulting in the final product. As stated by Garino and collaborators [27•], the allergenic risk would be associated with the consumption of a reasonable serving size, but the actual content of protein in a serving size is highly variable. In their study, the authors considered foods containing different percentages of mealworm protein, and, basing on the dose-response curve elaborated by Broekman and collaborators [52], they identified a possible eliciting dose. With the currently available data, it is not possible to define a general threshold dose, but authors suggest that a few milligrams of insect tropomyosin could be able to elicit a clinical response in shrimp sensitized individuals, and this dose is far lower than the whole hypothetical serving size [27•]. Spanjerserber and collaborators [87] propose an alternative quantitative risk assessment model based on probabilistic techniques, which returns a more exhaustive risk assessment and detailed information. The method permits the prediction of the possible severity of the reaction associated with the presence of a food allergen in a product and considers the allergen intake as a variable that influences the outcome of the prediction.

## Conclusion

The information available on the allergenicity of edible insects is still very limited. They can cross-react with other largely consumed foods like crustaceans but also with widespread invertebrate inhalant allergens like HDM. There is a great need for data deriving from studies based on subjects with confirmed allergy to edible insects. This will help to understand the way of sensitization and the possible cross-reactivity with other species. Indeed, it seems that the proteins responsible for cross-reactive phenomena are different from those playing a role in the primary sensitization to species like YMW. These studies should also include tests based on single purified allergens to allow the cross-inhibition experiments between, e.g., crustaceans (food), mite, and cockroach (inhalant), and between different insect species. In case several single allergens will be available, a component-resolved diagnosis (CRD) could be performed. Only by this kind of approach it will be possible to understand the frequency of sensitization, identify major or minor allergens, and assess the risk of allergic reactions due to IgE-cross reactivity.

The effects of processing should represent another aim of future research. In this case, the processing of more realistic model foods in which insects are combined with other food ingredients in complex matrices will help to understand the possible modifications of the epitopes and how this could impact the cross-reactivity and sensitization capacity of insect allergens.

## Abbreviations

AK: Arginine kinase
BAT: Basophil activation test
EFSA: European Food Safety Authority
GAPDH: Glyceraldehyde 3-phosphate dehydrogenase
HDM: House dust mite
HEXB1: Hexamerin 1B
HSP: Heat shock protein
IgE: Immunoglobulin E
SPT: Skin prick test
SW: Silkworm
TM: Tropomyosin
YMW: Yellow mealworm
